# Transcriptomic analysis identifies CXCL12 as a novel candidate gene for litter size in rabbits

**DOI:** 10.5713/ab.24.0640

**Published:** 2025-03-31

**Authors:** Zhiyuan Bao, Jie Yang, Jiali Li, Jiawei Cai, Pin Zhai, Pinyi Zhao, Bohao Zhao, Yang Chen, Xinsheng Wu

**Affiliations:** 1 College of Animal Science and Technology, Yangzhou University, Yangzhou, China; 2 Institute of Animal Science, Jiangsu Academy of Agricultural Sciences, Nanjing, China; 3 Joint International Research Laboratory of Agriculture & Agri-Product Safety, Yangzhou University, Yangzhou, China

**Keywords:** C-X-C Motif Chemokine Ligand 12 (CXCL12), JAK/STAT Pathway, Litter Size, Ovary, RNA-seq

## Abstract

**Objective:**

The ovary, as an important reproductive organ, tightly regulates the litter size of rabbits through its complex network of genes. This study aims to identify candidate genes related to litter size in rabbits through RNA sequencing and to analyze the regulatory effects of C-X-C motif chemokine ligand 12 (CXCL12) on granulosa cells (GCs).

**Methods:**

This study used ovarian transcriptome sequencing to identify differentially expressed genes between the ovarian tissues of New Zealand female rabbits with high (H) and low (L) litter sizes. In addition, a new candidate gene which was highly expressed in group H, namely the chemokine ligand CXCL12, was selected for further verification of biological functions.

**Results:**

The cell counting kit-8 assay and flow cytometry analysis showed that CXCL12 can promote GCs proliferation but inhibit their apoptosis. Furthermore, quantitative reverse transcription polymerase chain reaction and western blotting indicated that CRABP1 regulated genes (*PCNA*, *CCND1*, *CDK2*, *Bcl-2* and *Bax*) and proteins (CCND1, PCNA, Bcl-2 and Bax) related to cell cycle and cell proliferation. In addition, it can also regulate the expression levels of genes (*TAF4B*, *CITED1*, *WNT2*, *WNT10B*, and *HSD17B1*) and proteins (CITED1 and WNT10B) related to follicle development and litter size. Finally, it was found that CXCL12 targeted the CXCR4 receptor to activate the JAK/STAT signaling pathway.

**Conclusion:**

We utilized bioinformatics to screen 184 genes potentially associated with litter size in New Zealand female rabbits. Among these, CXCL12 plays a role in regulating the expression of cell cycle-related genes, promoting GCs proliferation. As a result, CXCL12 emerges as a promising candidate marker for selecting high litter size female rabbits in future breeding and production practices.

## INTRODUCTION

Litter size is a crucial economic factor in livestock production, but it also represents a complex attribute that is influenced by various dependent traits, such as ovulation rate, embryo survival rate, uterine capacity and environmental factors, amongst others [1]. Litter size varies between different breeding farms. Changing litter size using traditional breeding methods can be slow and has low heritability. The ovaries, as the primary reproductive organs in females, play a significant role in reproductive efficiency through ovulation. Therefore, investigating the different expression profiles of key ovarian genes can help elucidate the diversity in litter sizes [2]. Transcriptomics can study the mRNA transcription level, study the gene structure and function at the overall level, and study the deep transcriptional regulation to reveal the molecular mechanism of specific biological processes. Transcriptome analysis of mRNA expression levels in the ovaries of female rabbits with different litter sizes can help identify candidate genes involved in reproduction regulation. To date, RNA sequencing (RNA-Seq) has been used to study specific ovarian genes in livestock, especially to identify those related to litter size such as follicle-stimulating hormone receptor (*FSHR*), platelet-derived growth factor D (*PDGFD*), DNA methyltransferase 3β (*DNMT3B*) and nerve Growth Factor (*NGF*) [3]. However, the precise functions and molecular mechanisms of these genes are yet to be fully understood.

The chemokine ligand C-X-C motif chemokine ligand 12 (CXCL12), widely present in mammals and some primitive vertebrates, is known for directing chemotaxis and migration of immune cells [4]. It also participates in multiple physiological processes such as cell proliferation, cell migration, stem cell maintenance, and embryonic development by binding to the receptor CXCR4 or CXCR7. CXCL12 binding to CXCR4 is dependent on the N-terminal first eight amino acids of CXCL12, especially the first two amino acids Lys and Pro, in addition the N-terminal RFFESH amino acid sequence is responsible for the initial contact between CXCL12 and CXCR4 and induces conformational changes to activate the receptor [5]. The CXCL12/CXCR4 axis is crucial in tumor progression, especially due to its role in tumor angiogenesis, cancer cell metastasis and survival [6]. For instance, increased CXCL12 in rat mammary adenocarcinoma cells led to higher micro-vessel density and enhanced cancer cell invasiveness [7]. Similarly, in small cell lung cancer, the CXCL12/CXCR4 axis could promote integrin activation, thereby increasing drug resistance and promoting tumor cell growth [8], while in pancreatic cancer tissues, the expression of CXCL12 suggested that the CXCL12/CXCR4 axis could promote the proliferation, migration and invasion of pancreatic cancer cells through a paracrine mechanism [9]. CXCL12 also plays a significant role in the reproductive system. In particular, the CXCL12-CXCR4 chemokine-chemokine receptor pair is involved in various reproductive processes such as uterine natural killer cell recruitment, placental development, implantation as well as embryogenesis [10]. In addition, CXCL12 is not only expressed in follicles at different developmental stages, but is also secreted by primordial follicles [11]. In other cases, the application of CXCL12 in the *in vitro* maturation of sheep and pig oocytes was shown to increase the oocyte maturation rate and promote cumulus expansion [12]. Finally, reports suggest that CXCL12 could not only mitigate oxidative stress damage in human sperm by reducing the production of mitochondrial reactive oxygen species but also protect human sperm from cryoinjury [13].

However, in rabbit breeding and production, some New Zealand rabbits have low litter sizes, which seriously restricts production. In this study, the ovaries of high- and low-litter sizes producing female rabbits were used as samples, and differentially expressed genes (DEGs) were analyzed using RNA-Seq technology. However, despite these findings, the differences in CXCL12 expression between litter sizes are currently understood only at the transcriptome screening level, while its specific molecular regulatory mechanism remains unclear [14]. In this study, we believe that the high expression of CXCL12 in the ovaries of high-yielding mother rabbits suggests that it may be a candidate gene capable of increasing litter size. Therefore, this study aimed to identify the associations between CXCL12 and reproductive traits. It is expected that the findings will provide valuable transcriptional regulatory resources for understanding the mechanism of ovarian function in female rabbits, while also offering new insights into the role of litter size-related genes in determining mammalian litter size. This study aims to identify the main genes that control the reproductive traits of female rabbits and provide a molecular basis for the genetic improvement of reproductive traits of female rabbits.

## MATERIALS AND METHODS

### Animals and samples collection

For this study, the animal experiments were approved by the Animal Ethics Committee of Yangzhou University, China (approval No. 202212006), a total of six healthy and different families female New Zealand rabbits of similar age and parity were selected from the following two groups: an extremely high litter size group (H: n = 3) and an extremely low litter size group (L: n = 3), this was based on the average litter size after three births. Ovaries were collected from all female rabbits two months after the last parturition. The animals were anesthetized by injecting Zoteil-50 through the ear vein prior to collection of ovarian tissues. All animal experiments were performed under the review and supervision of the Animal Care and Use Ethics Committee of Yangzhou University.

### RNA extraction and sequencing

Trizol (Invitrogen, Burlington, ON, Canada) was used to extract total RNA from the collected ovarian tissues, with the RNA’s quality and quantity subsequently determined using an Agilent 2100 Bioanalyzer and a NanoDrop 1000 spectrophotometer (Thermo Scientific, Waltham, MA, USA), respectively. This was followed by library construction and sequencing at BGI Bioinformatics Technology Co., Ltd. (Shenzhen, China). Briefly, this involved mRNA enrichment with a polyA tail using OligodT magnetic beads after which the RNA was randomly fragmented, Reverse transcription was then performed using N6 primers, and the sequence end was filled, while the 5’ end was phosphorylated. The 3’ sticky end with a protruding “A” was also ligated to a bubble-shaped adapter containing a protruding “T” at the 3’ end. The resulting ligation product was polymerase chain reaction (PCR)-amplified using specific primers before being thermally denatured into single-stranded DNA. The latter was finally circularized using a bridge primer, with the resulting library of single-stranded circular DNA subsequently sequenced on the Illumina HiSeq 2500 platform (Illumina, San Diego, CA, USA).

### RNA-Seq analysis

Reads which contained adapters, were of low quality (more than 20% of <Q10 bases) and had more than 5% of unknown bases in the raw data were filtered using SOAPnuke (v1.4.0) and Trimmomatic (v0.36) software [15]. The resulting clean reads were then aligned to the ensembl *oryctolagus_cuniculus* reference genome sequence (version release-98) with HISAT (v2.1.0) [16] before calculating gene expression using Fragments Per Kilobase Millon Mapped Reads (FPKM). DESeq was also used for differential expression analysis between groups, with a |log_2_fold change| of ≥ 1 and a p-value of <0.05 selected as thresholds to screen for DEGs. Eventually, Gene Ontology (GO) annotation and Kyoto encyclopedia of genes and genomes (KEGG) pathway analysis were performed for all DEGs using the Database for Annotation [17].

### Immunofluorescence staining

Ovaries were fixed in 10% paraformaldehyde for 6 h, after which 5-μm thick sections were cut with a cryostat (Cryostar NX50; ThermoFisher Scientific) to obtain five sections from each ovary. These samples were then treated with 0.3% TritonX-100 (Beyotime) and 1% bovine serum albumin (Sigma, St. Louis, MO, USA) for 60 min prior to overnight incubation at 4°C with the following primary antibodies: anti-CXCL12 rabbit polyclonal antibody (1:250, Proteintech, Wuhan, China) and anti-CXCR4 rabbit polyclonal antibody (1:250, Proteintech). This was followed by a second incubation for 60 min at 37°C using goat anti-rabbit secondary antibody (1:100, Proteintech) before staining the paraffinized sections for 10 min using fluorescent dye 4’,6-diamidino-2-phenylindole (Beyotime) at room temperature. The cells were eventually visualized under a fluorescence microscope (Olympus CKX53; Olympus, Tokyo, Japan) and photographed. Each experiment was repeated at least three times.

### Plasmids and siRNA

The pcDNA3.1 (+) plasmid was used as a vector, while the ClonExpress II One Step Cloning kit (Vazyme, Nanjing, China) was used to construct the eukaryotic expression vector of CXCL12. In addition, one-step cloning primers (Supplement 1) were designed with the software DE Design v1.04 (Vazyme), with Nhe I and Xho I restriction sites added to the upstream and downstream primers, respectively. A small interfering RNA (siRNA) targeting CXCL12 was also designed and synthesized by Gema (GenePharma, Shanghai, China) (Supplement 2).

### Ovarian granular cell culture and transfection

A granulosa cells (GCs) culture was established in DMEM/F12 medium, supplemented with 10% fetal bovine serum (One ShotTM; Gibco, Waltham, MA, USA), using the immortalized cell line constructed in the current authors’ laboratory [18]. Separately, the plasmids or siRNAs were diluted with GIBCO Opti-MEM and after mixing with the LipofecCtamine3000 (Thermo Fisher) transfection reagent, a 15-min incubation was performed. Once the growth density of GCs reached 70%, they were seeded into a 24-well plate, and the transfection reagent was then added for GCs expression. The cells were further cultured at 37°C and under 5% CO_2_. The cells were eventually treated with 1 μm of MSX-122 (MedChemExpress, Shanghai, China) for 48 h to inhibit the binding of CXCL12 to its receptor CXCR4, thereby inhibiting pathway activity [19].

### RNA extraction and quantitative real-time polymerase chain reaction

Cells and samples of ovarian tissues were processed and added to a lysis solution. Each group included three biological replicates (n = 3), and each sample included three technical replicates. Total RNA was then extracted using the RNAsimple total RNA kit (TIANGEN, Beijing, China) according to the manufacturer’s instructions prior to synthesis of the corresponding cDNA with the HiScript II Q Select RT SuperMix (Vazyme). To determine the difference in the amount of other target genes after the change of CXCL12 gene, the Ct value of the target gene in the experimental sample was compared with the Ct value of the control sample, and the result was expressed as the ratio or difference multiple of the target gene amount in the experimental sample to the target gene amount in the control sample. This was followed by quantitative reverse transcription (qRT)-PCR, performed in a QuantStudio Design & Analysis machine (Applied Biosystems, Waltham, MA, USA), using the ChamQTM SYBR qPCR Master Mix (Vazyme). Each group consists of three biological replicates, with each replicate further comprising three technical replicates. In this case, glyceraldehyde-3-phosphate dehydrogenase (GAPDH) was used as the stable endogenous reference gene, and the relative gene expression level was eventually calculated using the 2^−ΔΔCT^ method [20]. The primer sequences used for this experiment are provided in Supplement 1.

### Automated protein analysis

After knocking down or overexpressing CXCL12, western blotting (WB) technology was used to detect the expression changes of related pathway proteins to determine the expression regulation relationship of the target gene activating or inhibiting the target protein and explore the signal pathway. WB was performed using the Wes^M^ automatic analyzer (Protein Simple, New York, NY, USA). For this purpose, cellular lysis was first achieved using RIPA buffer (PPLYGEN, Beijing, China), and the protein concentration was then determined using a bicinchoninic acid protein kit (Beyotime). These protein samples were standardized to a concentration of 0.5 μg/μL after which 3 μL of each were separated by gel electrophoresis. Protein detection was achieved using the following antibodies: anti-CCND1 mouse monoclonal antibody (1:250, Proteintech), anti-PCNA rabbit polyclonal antibody (1:250, Proteintech), anti-Bcl2 rabbit polyclonal antibody (1:250, Proteintech), anti-Bax rabbit polyclonal antibody (1:250, Proteintech), anti-CITED1 rabbit polyclonal antibody (1:50, Proteintech), anti-WNT10B mouse monoclonal polyclonal antibody (1:250, Proteintech), anti-CXCR4 mouse monoclonal polyclonal antibody (1:250, Proteintech), anti-phospho-JAK2 rabbit monoclonal polyclonal antibody (1:250, Abcam, Cambridge, UK), anti-JAK2 rabbit monoclonal polyclonal antibody (1:250, Abcam), anti-phospho-STAT1 rabbit polyclonal antibody (1:250, Proteintech), anti-STAT1 rabbit polyclonal antibody (1:250, Proteintech), anti-GAPDH mouse monoclonal antibody (1:2,500, Proteintech), 1:1,000 goat anti-rabbit secondary antibody IgG (Proteintech) and 1:1,000 goat anti-mouse secondary antibody IgG (Proteintech). We used each antibody after validation, so they all have good specificity [21–29]. The detected protein levels were eventually analyzed with the WES automatic protein analyzer system by following the manufacturer’s instructions [30].

### Cell proliferation and apoptosis

Cell counting kit-8 (CCK-8; Beytime) was used to assess the proliferation level of GCs over 48 h. For this purpose, the transfected GCs were first seeded into 96-well plates before adding 10 μL of CCK-8 solution into each well. After a 3-h incubation in a cell culture incubator, the optical density values at 0, 24 and 48 h were determined at 450 nm using the Infinite M200 Pro (Tecan, Männedorf, Switzerland). Apoptosis level was also detected using Annexin V-FITC/PI kit (Vazyme). In this case, 5 μL of Annexin V-FITC and PI staining solution was added to the cells in the dark at room temperature. This was followed by a 10-min incubation, after which 300 μL of binding buffer was added. The results were detected by flow cytometry (Becton Dickinson, Franklin Lakes, NJ, USA) and analyzed using CytExpert software (Shanghai, China), with the apoptosis rate eventually determined by counting the number of cells in the Q1-UR and Q1-LR areas.

### Statistical analysis

Each experiment was repeated at least three times, and the results were expressed as mean±standard deviation. The experimental and control groups were then compared using one-way analysis of variance and paired T-tests in IBM SPSS Statistics 25 (SPSS Inc., Chicago, IL, USA), the graphical representations were performed using the GraphPad Prism software (v10.0; GraphPad, Boston, MA, USA), with statistical significance was defined as * p<0.05, ** p<0.01, *** p<0.001.

## RESULTS

### RNA-Seq analysis

**Figure 1:**
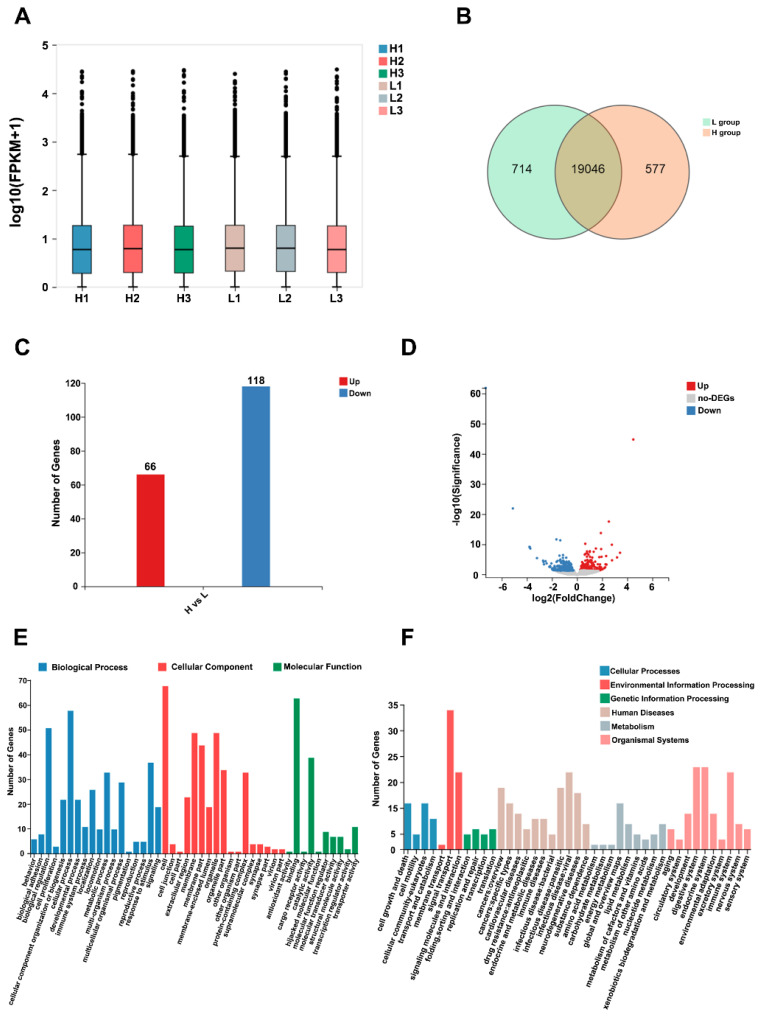
RNA-seq analysis of H vs L groups to identify of DEGs. (A) RSD plots of the different detected genes. (B) Venn diagram showing genes only expressed in the L group (green circle), only expressed in the H group (saffron yellow circle), and common to both groups (overlapping region). (C) DEGs identified between H and L groups. 66 up-regulated differentially expressed genes and 118 down-regulated differentially expressed genes were identified in the H group. (D) Volcano Plot of DEGs, Dot plots show each DEG between H and L. Red and blue dots represent significantly altered DEGs between H and L, respectively (p<0.05, fold change >1.5). (E) GO annotation of DEGs while the GO terms in the biological process included cellular process, biological regulation and response to biotic stimulus. (F) Annotation of KEGG pathways for DEGs revealed that the DEGs could be involved in signaling transduction, signaling molecules and interaction and the digestive system, amongst others. DEGs, differentially expressed genes; GO, gene ontology; KEGG, Kyoto encyclopedia of genes and genomes.

After quality control, it was found that the RNA integrity/quality (RIN/RQN) was above 8.0, as shown in Supplement 3. Therefore, the RNA samples were deemed as meeting the requirements for library construction. After sequencing, adapter removal and filtering of low-quality sequences, each sample yielded an average of 6.65 Gb of data for subsequent analysis. These data are available from the Sequence Read Archive (Bioproject: PRJNA283575). The average alignment rate of the sample compared to the genome as well as the average alignment rate of the compared gene set were 86.03% and 62.78%, respectively, while the Q30 value was above 87% (Supplement 4). Overall, the trend in gene expression was consistent across the six ovarian samples from the two groups (H and L) (Figure 1A), with 19,046 shared genes identified. However, 714 genes were also specifically expressed in the L group, while the expression of 577 genes was specific to the H group (Figure 1B). Of these, compared with the H group, 184 DEGs were subsequently screened out in the L group, and they included 66 up-regulated and 118 down-regulated genes (Figure 1C). Details regarding those DEGs are listed in Supplement 5. In addition, the volcano plot of differential genes (Figure 1D) clearly show the up- and down-regulation distribution of differential DEGs. Subsequent GO enrichment analysis found that the DEGs in the cellular component included cell, membrane and organelle, while the GO terms in the biological process included cellular process, biological regulation and response to biotic stimulus. Finally, for the molecular function, GO terms, such as binding and catalytic activity (Figure 1E), were enriched. Similarly, KEGG pathway analysis revealed that the DEGs could be involved in signaling transduction, signaling molecules and interaction and the digestive system, amongst others (Figure 1F). Our results indicate that the DEGs are involved in a wide range of regulatory functions in rabbit ovaries.

### Validation of differentially expressed genes by quantitative polymerase chain reaction

**Figure 2:**
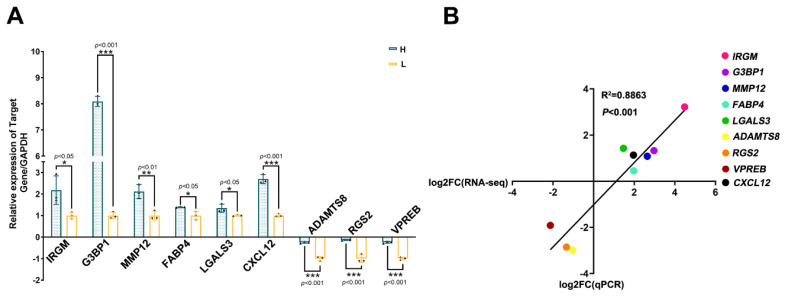
RT-qPCR-based verification the DEGs expression (A) positive correlation between RT-qPCR results and RNA-seq data (B). RT-qPCR was performed using gene-specific primers. RT-qPCR data were analyzed using 2−ΔΔCt for relative quantification. Data were expressed as mean±standard deviation (SD). Each experiment was repeated three times (Genes with asterisk differ significantly, the t test was used for the above analyses comparing two individual samples. * p<0.05; ** p<0.01; *** p<0.001). GAPDH, glyceraldehyde-3-phosphate dehydrogenase; RT-qPCR, real-time reverse transcription quantitative polymerase chain reaction; DEGs, differentially expressed genes.

qPCR analysis was performed using eight ovarian genes (immunity related GTPase M [*IRGM*], G3BP stress granule assembly factor 1 [*G3BP1*], matrix metallopeptidase 12 [*MMP12*], fatty acid binding protein 4 [*FABP4*], galectin 3 [*LGALS3*], ADAM metallopeptidase with thrombospondin type 1 motif 8 [*ADAMTS8*], the chemokine ligand CXCL12, regulator of G protein signaling 2 [*RGS2*]) and V-set pre-B cell surrogate light chain (*VPREB*) of groups H and L to validate the expression profiles obtained from RNA-Seq. Overall, the results were consistent with those of RNA-Seq (Figure 2A). Specifically, the relative fold change in gene expression, as obtained by qPCR, agreed with that of RNA-Seq. Furthermore, the expression of the eight genes, as detected by qRT-PCR, was positively correlated with that obtained from the RNA-seq data, with the linear correlation coefficient being 0.8863. A p<0.001 (Figure 2B). In all cases, the relative fold changes in gene expression in RNA-Seq and qPCR data were in the same direction. The results demonstrate the accuracy and authenticity of the ovarian RNA-Seq results. Furthermore, CXCL12, as a key molecule, has piqued our interest, prompting further investigation into its functions. In RNA-seq, the FPKM value of CXCL12 in the H array was 100.94, and the value in the L array was 49.366. The Q-value was 0.0000174. The table shows that it is significantly overexpressed in the ovaries of high litter size rabbits. qPCR-based detection of *CXCL12* expression in the ovaries of groups H and L, the expression of CXCL12 results showed high expression in group H (Figure 2A).

### Overexpression and knockdown of *CXCL12* in granulosa cells

**Figure 3:**
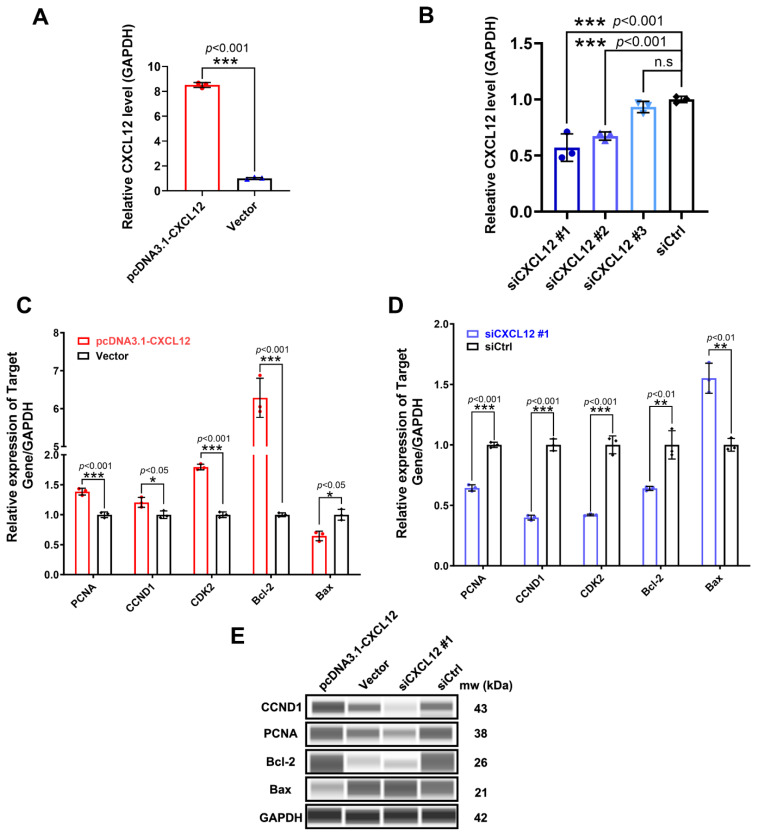
*CXCL12* overexpression and knockdown in GCs. (A,B) Changes in the expression of *CXCL12* after its overexpression and knockdown in GCs. (C,D) The mRNA expression level of *PCNA*, *CCND1*, *CDK2*, *Bcl-2* and *Bax* was detected after overexpressing or knocking down *CXCL12* in GCs. (E) The protein expression levels of CCND1, PCNA, Bcl-2 and Bax were detected after overexpressing and knocking down *CXCL12* in GCs. H, high-litter size group; L, low-itter size group; GAPDH was selected as the internal reference gene (The t test was used for the above analyses comparing two individual samples. * p<0.05; ** p<0.01; *** p<0.001; ns, not significant). GAPDH, glyceraldehyde-3-phosphate dehydrogenase; GC, granulosa cells.

*CXCL12* was overexpressed in GCs as shown in Figure 3A. Significant (p<0.0001) up-regulation of *CXCL12* expression was achieved in GCs using pcDNA3.1-CXCL12, while (Figure 3B). The siCXCL12 #1 was selected for subsequent experiments due to its high efficiency of interference. This result shows that we have successfully up-regulated and down-regulated the expression of CXCL12 in GCs. Markers of cell proliferation and cell cycle promotion include proliferating cell nuclear antigen (*PCNA*), Cyclin D1 gene (*CCND1*), Cyclin dependent kinase 2 (*CDK2*), BCL2 apoptosis regulator (*Bcl-2*) and BCL2 associated X (*Bax*) [31]. However, it was found that overexpressing *CXCL12* could significantly down-regulate *Bax* and up-regulate the expression of *PCNA*, *CCND1*, *CDK2* and *Bcl-2* (Figure 3C). In contrast, knocking down *CXCL12* significantly up-regulated *Bax* but down-regulated the expression of *PCNA*, *CCND1*, *CDK2* and *Bcl-2* (Figure 3D). Assessing the protein expression levels of these genes further confirmed that they were consistent with the mRNA expression trend (Figure 3E). It shows that CXCL12 can significantly promote GCs cycle and promote GCs proliferation.

### CXCL12 enhanced granulosa cells proliferation and inhibited their apoptosis

**Figure 4:**
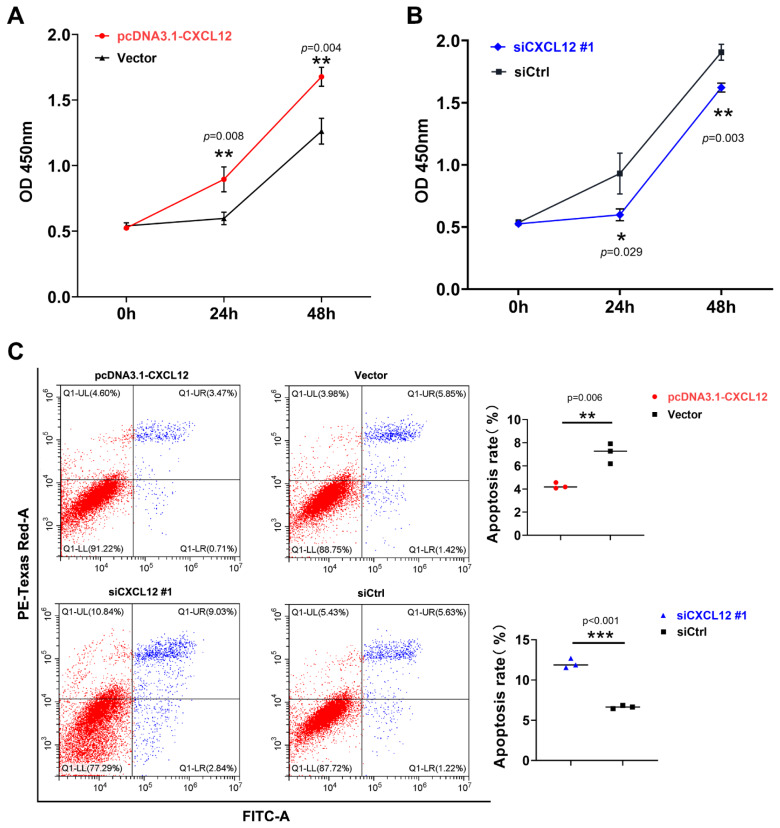
CXCL12 enhanced GCs proliferation and inhibited its apoptosis. The CCK-8 assay detected the proliferation of GCs after treatment with overexpressed (A) and knocked down (B) *CXCL12* for 0, 24 and 48 h. (C) Detection of GCs apoptosis after overexpressing or knocking down *CXCL12* (Statistical analyses were carried out using two-way ANOVA. The results of the significance test were significant after using Tukey's post hoc test * p<0.05; ** p<0.01; *** p<0.001). OD, optical density values; GC, granulosa cells.

The CCK-8 and FITC/PI method were used to detect the proliferation and apoptosis of GCs after overexpression and knockdown of *CXCL12*. The blue dots, the Q1-UR and Q1-LR regions, represent regions of apoptotic cell staining. The red dots Q1-UL represent cell fragments, and Q1-LL represent viable cells. Therefore, the ratio of the two regions added represents the proportion of apoptotic cells in this group. This was used to analyze the level of apoptosis in different groups. The results showed that overexpressing *CXCL12* significantly increased the proliferation rate of GCs at 24 and 48 h (p<0.01), while significantly inhibiting their apoptosis (p<0.01) (Figures 4A, 4C). On the other hand, knocking down *CXCL12* significantly inhibited the proliferation of GCs at 24 and 48 h (p<0.01) (Figure 4B), but significantly increased their apoptosis rate (p<0.01) (Figure 4C). These results suggest that CXCL12 promotes GCs proliferation and inhibits cell apoptosis.

### CXCL12 interacts with litter size- and ovary function-related genes

**Figure 5:**
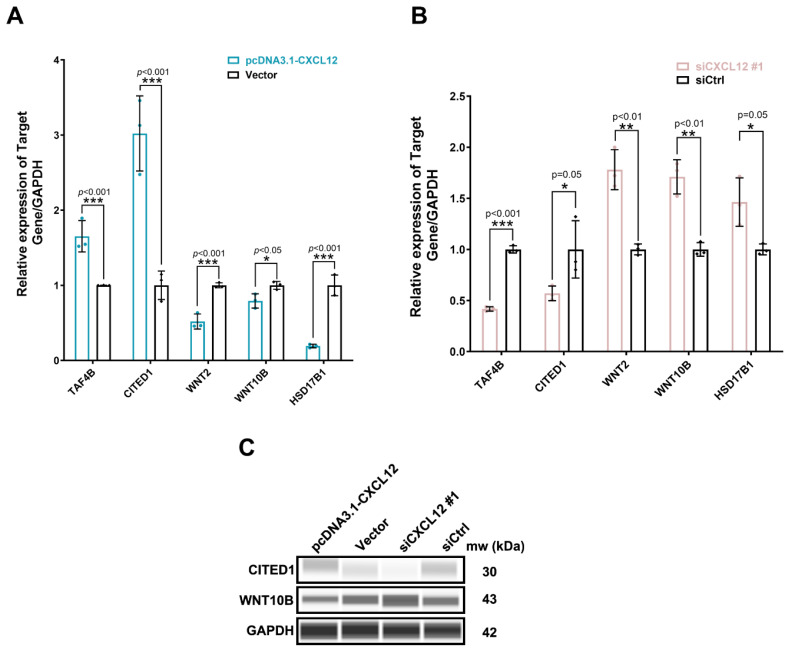
*CXCL12* regulated the expression of genes and proteins related to litter size and ovarian functions. The mRNA expression level of *TAF4B*, *CITED1*, *WNT2*, *WNT10B* and *HSD17B1* were detected after overexpression (A) and knockdown (B) of *CXCL12* in GCs. (C) The protein expression levels of CITED1 and WNT10B were detected after overexpression and knockdown of CXCL12 in GCs (The t test was used for the above analyses comparing two individual samples. * p<0.05; ** p<0.01; *** p<0.001). GAPDH, glyceraldehyde-3-phosphate dehydrogenase; GC, granulosa cells.

WB and qRT-PCR were used to further verify the regulatory mechanism of CXCL12 in GCs. Analysis showed that the pcDNA3.1-CXCL12 overexpression plasmid and siCXCL12 #1 interference sequence were successfully transferred into GCs, with the expression levels of *TAF4B*, *CITED1*, *WNT2*, *WNT10B* and *HSD17B1* subsequently detected by qRT-PCR. Overexpressing *CXCL12* significantly increased the expression level of *TAF4B* and *CITED1* (p<0.0001), but significantly decreased that of *WNT2*, *WNT10B* and *HSD17B1* (Figure 5A). However, the expression trends displayed opposite effects when *CXCL12* was knocked down (p<0.001) (Figure 5B). In addition, at the protein level, it was found that CXCL12 overexpression significantly increased the expression levels of the CITED1 and WNT10B proteins, while knockdown significantly inhibited their expression (Figure 5C). This indicates that CXCL12 can regulate the expression of reproduction-related genes and plays an important role in regulating the litter size of female rabbits.

### CXCL12 triggers the CXCR4 receptor and activates the JAK/STAT pathway

**Figure 6:**
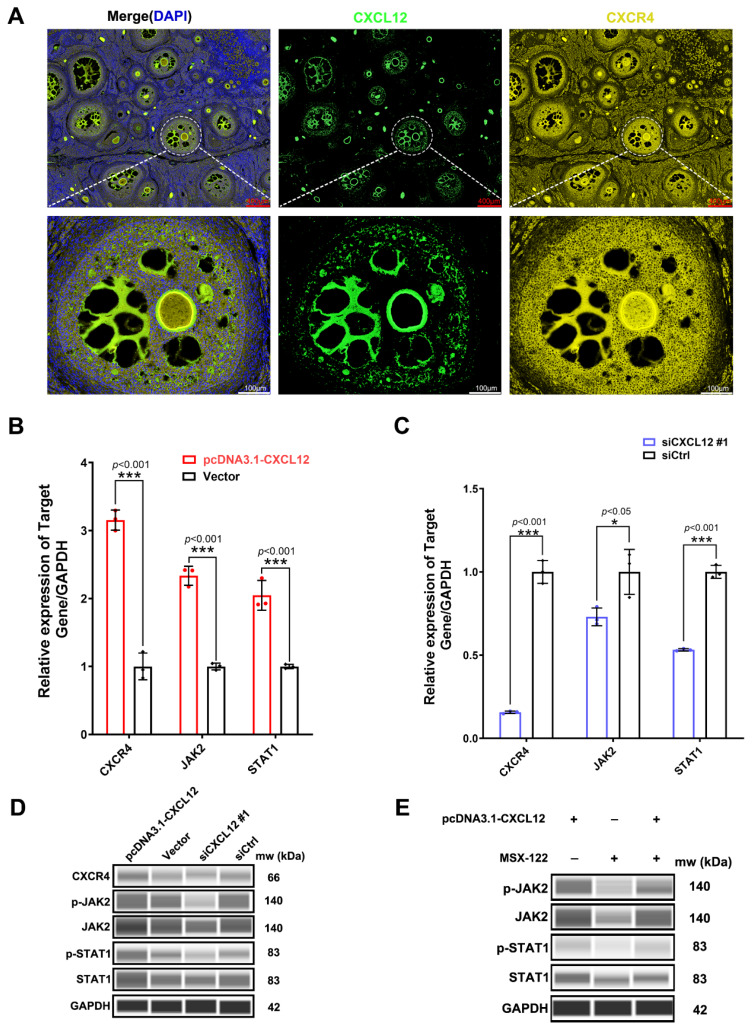
CXCL12 and its receptor CXCR4 were expressed and localized in the ovary, with their binding activating the JAK/STAT signaling pathway. (A) Immunofluorescence staining of ovarian follicles revealed that CXCL12 was co-localized with its receptor CXCR4 in ovarian follicles. (B) *CXCL12* overexpression significantly promoted the expression of *CXCR4*, *JAK2* and *STAT1*. (C) Knocking down *CXCL12* significantly inhibited the expression of *CXCR4*, *JAK2* and *STAT1*. (D) *CXCL12* overexpression in GCs promoted the protein expression of CXCR4, JAK2 and STAT1 as well as the phosphorylation of JAK2 and STAT1. When *CXCL12* was knocked down, the results were reversed. (E) MSX-122 inhibitor-based treatment further confirmed that CXCL12 promoted the total protein and phosphorylation levels of JAK2 and STAT1 (The t test was used for the above analyses comparing two individual samples. * p<0.05; ** p<0.01; *** p<0.001).

Mature CXCL12 binds CXCR4 receptors with high affinity to activate downstream pathways [32]. In this study, CXCL12 and its receptor CXCR4 were first co-expressed in ovarian follicles, with the expressions confirmed by immunofluorescence. They were also expressed in GCs and oocyte membranes as well as in the follicular antrum, In addition, the follicular antrum also contained abundant staining results, suggesting that the follicular fluid was also filled with abundant expression. (Figure 6A). In this case, *CXCL12* overexpression significantly promoted the expression of CXCR4, JAK2 and STAT1 (Figure 6B), while *CXCL12* knockdown significantly inhibited their expression (Figure 6C). The overexpression of *CXCL12* in GCs also promoted the protein expression of CXCR4, JAK2 and STAT1 as well as the phosphorylation of the latter two. In contrast, knocking down *CXCL12* had the opposite effects (Figure 6D). Finally, the use of MSX-122 to prevent CXCL12 from binding to CXCR4 significantly inhibited the total protein and phosphorylation of JAK2 and STAT1 (Figure 6E), but overexpressing *CXCL12* reduced those inhibitory effects of MSX-122 (Figure 6F). This indicates that CXCL12 triggers the CXCR4 receptor and activates the JAK/STAT pathway.

## DISCUSSION

The ovary is a crucial reproductive organ in female mammals as it is responsible for the secretion of reproductive hormones as well as the maturation of oocytes. Therefore, its function directly impacts the fertility of female animals [33]. A complex transcriptional network, consisting of both coding and non-coding genes, tightly regulates ovarian function. Although researchers have employed various methods, such as physiology, reproduction, genetic markers, single-omics and candidate gene methods, to investigate the mechanisms behind high litter size traits, the precise regulatory mechanisms remain unclear, thereby necessitating further in-depth research [34]. Therefore, in this study, ovarian tissues from high-and low-producing female rabbits were selected for RNA-seq analysis to identify differentially expressed mRNAs. All experimental animals were from the same farm with the same environment, so the effect of the environment on litter size was negligible. Although the sample size of this study met the requirements of at least three biological replicates and the reproducibility between different groups was good, there are still certain limitations. In future studies, the sample size should be further expanded to facilitate better research results in the future.Analysis of the RNA-seq data subsequently revealed that CXCL12 was significantly expressed in the H group of New Zealand female rabbits, hence suggesting that this gene may play a regulatory role in rabbit reproductive performance. Relevant transcriptomic data show that CXCL12 was highly expressed in bovine fallopian tube and endometrial tissues [35]. Analysis of transcriptome data showed that CXCL12 and its receptor CXCR4 were highly expressed in reproductive system tissues as well as in *in vivo* embryos, suggesting that the CXCL12-CXCR4 axis may have a universal function in the female mammalian reproductive system [12]. In the mammalian reproductive system, chemokines are often involved in multimodal events closely related to the establishment, maintenance, and regression of fertility, It has been shown that Uterine injection of CXCL12 increased the pregnancy rates in a mouse model of endometriosis [36]. The expressions of CXCL12 in ovarian GCs of PCOS rats were decreased, and the apoptosis rate was increased. In human KGN cells, CXCL12 regulates the expression of BAX, BCL2 and cleaved CASP3 through CXCR4 and CXCR7-mediated signaling, thereby inhibiting cell apoptosis [11]. In addition, we found that CXCL12 promoted GCs proliferation and inhibited apoptosis *in vitro*. In other cases, the application of CXCL12 in the *in vitro* was shown to increase the oocyte maturation rate and promote cumulus expansion [37]. The essence of this is granule cell proliferation, which is consistent with our results. To further understand the role of CXCL12 in the reproduction process, its expression was up-regulated, with the results subsequently showing a significant increase in the expression of TAF4B and CITED1 as well as a significant decrease in the expression of WNT2, WNT10B and HSD17B1. However, after knocking down CXCL12, it was found that the expression levels of the above genes were opposite to those observed during overexpression. Mounting evidence further suggests that TAF4B, CITED1, WNT2, WNT10B and HSD17B1 impact female fertility in animals. For instance, mice, with the endogenous TAF4B knocked out, are infertile, while TAF4B mRNA and protein expression are almost exclusively present in germ cells of mouse embryonic ovaries. Furthermore, TAF4B-deficient ovaries display abnormalities such as delayed germ cell cyst rupture, reduced ovarian reserve as well as excessive depletion of perinatal germ cells [38,39]. HSD17B1, widely expressed in rodent and human ovarian GCs, is mainly involved in the conversion of estrogen into more biologically active estradiol [40] However, during the luteinization process in rats, its expression is significantly down-regulated [41]. In addition, the association between HSD17B1 SNP and litter size has been investigated as a potential molecular marker for increasing litter size in pigs [42]. Finally, CITED1, as a key site that affects transcriptional function and regulates the germ cell cycle, plays an important role in influencing female fertility [43]. Altogether, these findings suggest that CXCL12 as well as the TAF4B, CITED1 and HSD17B1 genes are interlinked and jointly regulate transcription and cell cycle. It is well established that WNT2 and WNT10B are key factors in the canonical WNT signaling pathway, with various studies further reporting that this pathway typically plays a positive role in follicular development [44]. However, interestingly, CXCL12 significantly reduced their mRNA levels, Moreover, the ability of CXCL12 to act through the canonical WNT signaling pathway was assessed. Blockade of CXCL12/CXCR4 signaling inhibits intrahepatic cholangiocarcinoma and breast cancer progression and metastasis via inactivation of canonical Wnt pathway [45,46]. However, in our study that we have a different finding. In this case, it was found that the gene only affected WNT2 and WNT10B expression but not that of other key factors in the canonical wnt signaling pathway (data not shown). Therefore, it was speculated that CXCL12 may act through the non-canonical WNT signaling pathway, although the specific mechanism requires further investigation. Therefore, we continued to explore the possible signaling pathways affected by CXCL12. Additionally, it was found that CXCL12 can regulate the expression levels of total and phosphorylated proteins of JAK and STAT1. Using pathway inhibitors, it was further found that CXCL12 can target the CXCR4 receptor to activate the JAK/STAT signaling pathway. The latter also plays a crucial role in influencing litter size. Research also shows that the JAK-STAT signaling pathway is important in GnRH neurons and thus, it participates in the reproductive process [47]. This study confirmed that CXCL12 can target the CXCR4 receptor to activate the JAK/STAT signaling pathway. By identifying the key genes affecting litter size, rabbit breeders can more specifically select breeding rabbits with high litter potential for breeding in practical production, thus rapidly improving the reproductive ability of the whole population and making long-term genetic improvement plans. In addition, further studies on gene function, improvement of multi-trait genetic evaluation system and interdisciplinary cooperation are needed in the future.

## CONCLUSION

**Figure 7:**
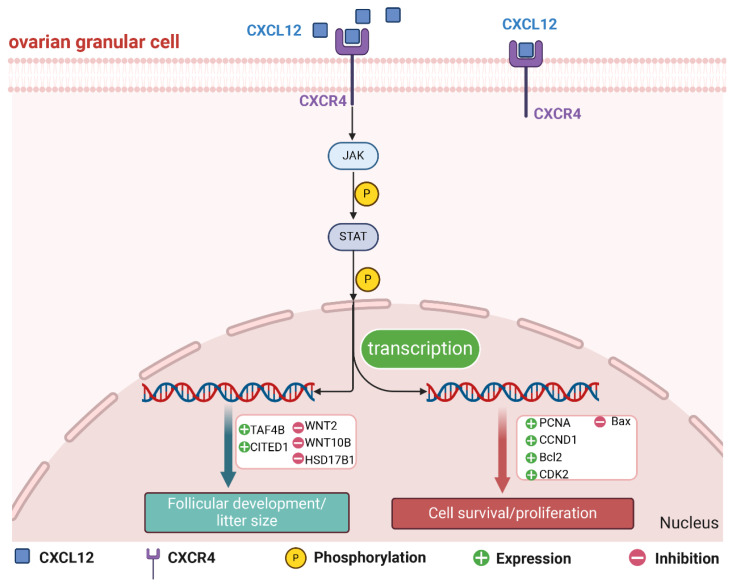
CXCL12 targets the receptor CXCR4 to activate the JAK/STAT signaling pathway and regulate the expression of related genes.

A total of 184 ovarian DEGs related to litter size were identified. *CXCL12* can regulate the expression of cell cycle-related genes (*PCNA*, *CCND1*, *CDK2*, *Bcl-2*, and *Bax*) and proteins (PCNA, CCND1, Bcl-2, and Bax) to promote GCs proliferation and inhibit its apoptosis. In addition, it also regulated genes (*TAF4B*, *CITED1*, *WNT2*, *WNT10B* and *HSD17B1*) and proteins (CITED1 and WNT10B) that were linked to litter sizes and follicular development. Overall, the CXCL12/CXCR4 axis works by activating the JAK/STAT signaling pathway (Figure 7).

## SUPPLEMENTARY MATERIAL

**Supplement 1.** Primer sequences used in this study for qRT-PCR

**Supplement 2.** Sequences of siRNA

**Supplement 3.** RNA quality of sequencing samples

**Supplement 4.** Results of sequencing data filtering and comparison

**Supplement 5.** Detailed list of differentially expressed genes
