# Supplementary material for: Transcriptomic analysis identifies CXCL12 as a novel candidate gene for litter size in rabbits
Source: Anim Biosci. 2025 Mar 31;39(1):240640. doi: 10.5713/ab.24.0640 (PMC12754513; doi:10.5713/ab.24.0640)
Supplement: Supplementary file 1 [file ab-24-0640-Supplementary-1.pdf]

Supplement 1. Primer sequences used in this study for qRT-PCR

Gene	Sequence (5'-3')
GAPDH	F: TCACCATCTTCCAGGAGCGA<br>R: CACAATGCCGAAGTGGTCGT
PCDNA3.1-CXCL12	F: gggagaccc*aagctggctagc*<br>ATGGACGCCAAGGTTGTCGCCGTGC<br>R: aacgggccct*ctagactcgag*<br>CTAGTTTTTCCTTTTCTGGGCAGCC
CXCL12	F: GTTGTCGCCGTGCTGGCCCT<br>R: CACACTTGTCGGTTGTTGCT
IRGM	F: ACAAAGCAGCCAGTTCCCAT<br>R: AGATGTCTCTGGCAGGCAAC
G3BP1	F: GCCGCGCAGGCAAAT<br>R: AGCAGGGGACTAGGCTTCTC
MMP12	F: ACAAGCCTCGATGTGGAGTG<br>R: TCCTCACGCTTCATGTCTGG
FABP4	F: AAGTCACCGCAGATGACAGG<br>R: CACCACCAGTTTATCGCCCT
LGALS3	F: CCAAGTGGTGCTGGAGCTTA<br>R: ACCGTGCCCACAATCGTTAT
ADAMTS8	F: ACAGTCCCTGGTGAGGTCTT<br>R: CGTTGGTGGTTGCTCTCTCT
RGS2	F: GGATTGGAAAACACG<br>R: TAAAAAAGCCCTGAA
VPREB	F: CCCTGCGCTTGATTTTCGG<br>R: ATTGCTGAGCGAGGTGGAAT
PCNA	F: AGCCTCGCGAAGAAGTTTCA

	R: GCAGGCCTCGTTGATGAGAT
CCND1	F: AACACGGACGTGGATTGTCT<br>R: TGGTCCAGTTCATCCTCCGA
Bcl-2	F: TTCTGAAAGGACGGGTCAGC<br>R: CCAAGGAACCCTTCAAGGCA
Bax	F: CGTTGTCGCCCTGTTTTACT<br>R: TTTGCCACGTGGGGGTCCCG
CDK2	F: AGTGGGCAGACCCAAGACTA<br>R: AAGCAATGGATGGGAGGGAG
HSD17B1	F: GAGCTTCAAAGTGTACGCCAC<br>R: TCCGAGTCCCTTACGTCCA
WNT10B	F: ATCGGCTCAGGTCCAAACTG<br>R: CATGGTTACAGCCACCCCAT
WNT2	F: CGGACGCAAGGGGGTTAATA<br>R: CCCTGGAGGAGTCACCTGTA
CITED	F: GCGCCGGTTTATCCAACTTC<br>R: GGTTGGCATCCTCCTTCACA
TAF4B	F: CGTGACAAGAGCTGAGACCA<br>R: GTGAGCTAGAATTCGGCACG
CXCR4	F: ACTGGCATAGTGGGCAATGG<br>R: CCAGAAGGGAAGCGTGATGA
JAK2	F: CCTTCTAGGGAGGGCCCAG<br>R: CCGTGGAGATTTTCAGGGCA
STAT1	F: CTTTCGTTTTCCAGGGCAGG<br>R: GAGTCGACGACGGTGTCTG

Note: The underlined italics for the enzyme cutting site. F:forward primer; R: reverse primer; F, forward primer; R, reverse primer
