# Supplementary material for: Transcriptomic analysis identifies CXCL12 as a novel candidate gene for litter size in rabbits
Source: Anim Biosci. 2025 Mar 31;39(1):240640. doi: 10.5713/ab.24.0640 (PMC12754513; doi:10.5713/ab.24.0640)
Supplement: Supplementary file 2 [file ab-24-0640-Supplementary-2.pdf]

Supplement 2. Sequences of siRNA

Name	Sequence (5′-3′)
siCXCL12 #1	S: GGCAGGUGAUAAGUGACUUTT<br>AS: AAGUCACUUAUCACCUGCCTT
siCXCL12 #2	S: GCAUUGAUCCGAAGCUAAATT<br>AS: UUUAGCUUCGGAUCAAUGCTT
siCXCL12 #3	S: GCCAACGUCAAGCAUCUCATT<br>AS: UGAGAUGCUUGACGUUGGCTT
siCtrl	S: UUCUUCGAACGUGUCACGUTT<br>AS: ACGUGACACGUUCGGAGAATT

Note: S: sense; AS: antisense; siCtrl: negative control.
