# Supplementary material for: Transcriptomic analysis identifies CXCL12 as a novel candidate gene for litter size in rabbits
Source: Anim Biosci. 2025 Mar 31;39(1):240640. doi: 10.5713/ab.24.0640 (PMC12754513; doi:10.5713/ab.24.0640)
Supplement: Supplementary file 3 [file ab-24-0640-Supplementary-3.pdf]

Supplement 3. RNA quality of sequencing samples

Group	Sample ID	concentration (ng/μL)	Volume (μL)	total quantity(μg)	RIN/RQN
H	H1	701.5	60	42.090	8.7
	H2	735.8	60	44.148	9.4
	H3	863.3	60	51.798	8.8
L	L1	1441.1	60	86.466	8.4
	L2	643.4	60	38.604	8.1
	L3	564.1	60	33.846	8.8

Note: RIN/RQN: RNA integrity/quality numbers.
