# Supplementary material for: Transcriptomic analysis identifies CXCL12 as a novel candidate gene for litter size in rabbits
Source: Anim Biosci. 2025 Mar 31;39(1):240640. doi: 10.5713/ab.24.0640 (PMC12754513; doi:10.5713/ab.24.0640)
Supplement: Supplementary file 4 [file ab-24-0640-Supplementary-4.pdf]

Supplement 4. Results of sequencing data filtering and comparison

Sample name	TRR (M)	TCR (M)	TCB (G)	Clean Reads Q20 (%)	Clean Reads Q30 (%)	Clean Reads Ratio (%)
H1	49.08	44.1	6.61	96.41	87.63	89.85
H2	49.08	44.21	6.63	96.56	88.06	90.08
H3	49.08	44.08	6.61	96.61	88.22	89.82
L1	50.83	45.77	6.87	96.39	87.5	90.05
L2	49.08	43.86	6.58	96.44	87.8	89.36
L3	49.08	43.91	6.59	96.26	87.23	89.46

Note: TRR: Total Raw Reads; TCR: Total Clean Reads; TCB: Total Clean Bases.
