# Supplementary material for: Transcriptomic analysis identifies CXCL12 as a novel candidate gene for litter size in rabbits
Source: Anim Biosci. 2025 Mar 31;39(1):240640. doi: 10.5713/ab.24.0640 (PMC12754513; doi:10.5713/ab.24.0640)
Supplement: Supplementary file 5 [file ab-24-0640-Supplementary-5.pdf]

Supplement 5. Detailed list of differentially expressed genes

Gene ID	Gene name	l FPKM	h FPKM	log2(h/l)	Qvalue(l-vs-h)	Pvalue(l-vs-h)
ENSOCUG00000028024.3	IRGM, LRG47	0.84	24.153	4.479866	1.74E-45	1.71E-49
ENSOCUG00000023781.2	CNOT7_8, CAF1, POP2	0.046	4.426	3.421978	6.34E-08	5.95E-11
BGI_novel_G001487	UCHL1	0	13.986	3.186051	0.00000224	3.42E-09
BGI_novel_G000079	GP2	0.12	2.056	2.786239	0.0000202	3.99E-08
ENSOCUG00000022069.2	G3BP1	1.706	14.913	2.772115	1.39E-10	6.18E-14
ENSOCUG00000024432.2	IFI47、KRAB	3.316	35.313	2.534468	2.89E-18	5.69E-22
ENSOCUG00000003082.4	P2RX3	0.38	3.606	2.347731	0.000529712	0.0000227
BGI_novel_G000818	GP2	0.246	1.5	2.317993	0.000000775	9.94E-10
BGI_novel_G000011	WDR32	0.46	3.596	2.267303	0.000426838	0.00000177
ENSOCUG00000008303.4	MMP12	0.856	11.753	2.244119	0.003987867	0.0000299
ENSOCUG00000021978.2	CENPQ	0	1.27	2.177027	0.007904437	0.0000799
BGI_novel_G001627	GP2、KRAB	0.033	0.703	2.03972	0.016663958	0.000219303
ENSOCUG00000005061.3	GST, gst	0.066	1.176	1.984611	0.021575342	0.000306986
ENSOCUG00000022596.2	adk, AK	0.923	4.02	1.951231	0.0000014	1.93E-09
BGI_novel_G002715		0.133	1.15	1.92135	0.021545133	0.000301806
BGI_novel_G002516		0.453	1.806	1.920407	3.84E-09	2.66E-12
ENSOCUG00000006285.3	HCST, DAP10	6.093	30.643	1.914632	0.004177155	0.000033
ENSOCUG00000015054.4	UNC5	0.82	3.113	1.89835	1.85E-14	4.55E-18
BGI_novel_G001349		0.07	1.836	1.833895	0.041147872	0.000864605
ENSOCUG00000026122.2	SF3B4, SAP49	1.413	7.823	1.831929	0.016438498	0.000210002
ENSOCUG00000027815.3	FCGR3, CD16	0.31	1.77	1.829601	0.026406207	0.000424604
ENSOCUG00000016469.3	MLANA, MART1	2.873	11.713	1.82844	0.000464744	0.00000195
BGI_novel_G001477		0.21	0.963	1.821936	0.00357464	0.0000261
BGI_novel_G000819		0.23	1.193	1.804461	0.015594177	0.000191524
BGI_novel_G001125	GP2、KRAB	0.446	1.96	1.793696	0.0027053	0.0000183
ENSOCUG00000017453.3	CES2	0.38	1.73	1.77171	0.01576376	0.000195161
ENSOCUG00000007445.4	FABP4, aP2、RBM16, SCAF8、FABP3	20.756	84.693	1.77148	0.00000178	2.63E-09
BGI_novel_G000378	FCRL, IRTA, CD307、LY9, CD229、CD84	0.613	2.926	1.70896	0.026755278	0.000437874
ENSOCUG00000004314.4	TRPM1	0.363	1.656	1.666983	0.038944835	0.000791421
ENSOCUG00000012592.4	ASB5	3.01	10.856	1.651528	0.000416526	0.00000164
ENSOCUG00000013423.4	STEAP3, TSAP6	0.64	2.336	1.619855	0.006258592	0.0000577
BGI_novel_G002540	GP2	0.326	1.18	1.561397	0.016663958	0.000219008
BGI_novel_G000817	GP2	0.246	1.006	1.559052	0.045020349	0.001003709
BGI_novel_G000232	MTG1	16.08	45.403	1.501813	2.51E-09	1.49E-12
BGI_novel_G001121		0.963	2.993	1.477186	0.004286322	0.0000342
ENSOCUG00000009659.3	LYVE1	5.143	14.12	1.447628	0.0000574	0.00000015
ENSOCUG00000002864.4	BLNK	3.273	9.61	1.374257	0.021719916	0.000316785
ENSOCUG00000026331.2	CBR1	4.913	13.343	1.363889	0.002881495	0.0000196
ENSOCUG00000015138.4	COL4A	5.126	13.42	1.347398	0.000693008	0.00000318
ENSOCUG00000001607.4	PRSS21、PRSS33	0.72	2.09	1.343234	0.048700682	0.001143411
ENSOCUG00000004172.4	VSIG4, CRIg	2.18	6.126	1.337088	0.000709923	0.00000329
ENSOCUG00000005726.4	RP-L17, MRPL17, rplQ	69.603	173.383	1.322211	2.95E-08	2.48E-11
ENSOCUG00000001490.4	IFNLR1, IL28RA	0.79	2.153	1.315399	0.041721527	0.000891063
BGI_novel_G000893		1.713	4.34	1.301179	0.00076064	0.0000036
ENSOCUG00000008629.4	KCNK2, K2P2.1	23.786	52.563	1.263568	0.000134419	0.000000431
ENSOCUG00000004890.4	SLC9A2, NHE2	3.6	8.966	1.215918	0.021607051	0.0003112
ENSOCUG00000005374.4	C7、CARD6	28.24	65.213	1.215584	0.00000249	3.93E-09
BGI_novel_G001124	GP2	0.973	2.283	1.203919	0.00260389	0.0000175
ENSOCUG00000011697.4	VCAM1, CD106	12.683	29.32	1.181772	0.000234485	0.000000844
ENSOCUG00000002924.4	LGALS3	373.813	862.313	1.168974	0.001235666	0.00000622
ENSOCUG00000015143.4	COL4A	5.61	12.85	1.160231	0.004033688	0.0000308
ENSOCUG00000004995.4	LOXL2_3_4、AL1	4.45	9.3	1.155868	0.000019	3.66E-08
ENSOCUG00000016385.4	CADM3, IGSF4B, NECL1, TSLL1	2.263	5.106	1.137835	0.00561142	0.0000507
ENSOCUG00000015938.4	CCDC64、GOLGB1、SYCP1、SMC1、PCNT、TCHH、C	5.833	12.65	1.134733	0.0000805	0.00000023
BGI_novel_G000078	GP2	0.716	1.636	1.127342	0.039588987	0.000825358
ENSOCUG00000006500.4	SLC4A4, NBC1	18.02	38.836	1.105114	0.0000925	0.000000274
ENSOCUG00000031964.1	C3	9.22	20.21	1.103573	0.006676761	0.0000639
BGI_novel_G000892		4.306	9.25	1.068468	0.027883788	0.000468993
ENSOCUG00000029076.2	CXCL12、FMN2	49.366	100.94	1.060355	0.0000174	3.26E-08
BGI_novel_G000177	CLCA3_4、V2R	2.266	4.773	1.059062	0.021545879	0.00030368
BGI_novel_G000233	MTG1	2.92	6.22	1.036935	0.041252392	0.000872448
ENSOCUG00000022690.3	HADHB	3.51	7.303	1.032873	0.026829788	0.000444649
BGI_novel_G000255	GCH1, folE、 dut, DUT、OLFR	7.83	16.166	1.032828	0.002130906	0.0000132
ENSOCUG00000006098.4	NOTCH1	9.02	18.036	1.03275	0.00000144	2.06E-09
ENSOCUG00000017780.4	EDNRB	3.563	7.06	1.018145	1.94E-08	1.44E-11
ENSOCUG00000001029.4	CDH17	5.883	10.56	1.008737	3.53E-08	3.13E-11
BGI_novel_G001597		1.673	0.776	-1.02009	0.00034126	0.00000128
BGI_novel_G001034	ANO5, GDD1, TMEM16E	4.493	2.03	-1.02551	0.004198539	0.0000333
ENSOCUG00000005228.4	NKD	9.066	4.126	-1.03037	0.0000571	0.000000146
ENSOCUG00000013668.4	LPPR1_2_5	4.316	1.903	-1.03064	0.010232771	0.000112553
ENSOCUG00000031274.1		7.066	3.16	-1.03329	0.005152807	0.0000455
ENSOCUG00000000074.2	HMGB2	127.22	55.046	-1.03773	0.03352409	0.00063331
ENSOCUG00000014236.4	NUP210, GP210	7.596	3.436	-1.03805	0.000341878	0.0000013
ENSOCUG00000016390.4	WISP1	15.063	6.77	-1.04165	0.002085098	0.0000128
ENSOCUG00000015523.4	RSF1、EEA1、SPN1, IWS1、RIF1、ZC3H13	7.706	3.38	-1.04273	0.01405391	0.000166368
BGI_novel_G001366	MAP4K1, HPK1	4.63	2.023	-1.05301	0.022941851	0.000349492
ENSOCUG00000008043.4	ERC2, CAST	1.006	0.44	-1.05383	0.038367644	0.000772122
ENSOCUG00000034817.1	PARP、SYN、DNAH、 GNAO, G-ALPHA-O、CLCA3_4	5.126	2.266	-1.06802	0.00034126	0.00000127
ENSOCUG00000001208.4	RXFP1, LGR7	1.983	0.773	-1.07423	0.001681298	0.00000928
ENSOCUG00000030106.1	PARP、SYN、DNAH、 GNAO, G-ALPHA-O、CLCA3_4	1.956	0.83	-1.07538	0.032215571	0.000596944
ENSOCUG00000016954.4	TTN	1.963	0.856	-1.07919	0.001901626	0.0000112
ENSOCUG00000028094.2	PRC1	9.066	3.95	-1.08191	0.039053953	0.000797491
BGI_novel_G002480	CANT1	10.833	4.656	-1.08283	0.006754411	0.0000653
ENSOCUG00000013450.4	MYH	52.443	23.113	-1.08401	0.0000767	0.000000212
ENSOCUG00000007574.4	KLK7, PRSS6	10.106	4.266	-1.08481	0.042205181	0.000903475
BGI_novel_G002438	SYN、DNAH	2.556	1.116	-1.09379	0.00030173	0.0000011
BGI_novel_G000144		8.19	3.586	-1.10243	0.0000264	5.72E-08
ENSOCUG00000017210.3	CBX6	16.563	6.99	-1.1058	0.004043806	0.0000311
ENSOCUG00000029486.2	GST, gst	277.016	114.733	-1.1061	0.012620999	0.00014567
ENSOCUG00000008699.4	COL22A	3.956	1.476	-1.11099	0.031307238	0.000558255
ENSOCUG00000008011.4	ABCA8、ABCA9	13.41	5.943	-1.11216	0.032658999	0.000610524
ENSOCUG00000009833.4	SLC24A3, NCKX3	2.22	0.936	-1.11504	0.004934595	0.0000417
ENSOCUG00000011464.4	CLDN	9.666	3.906	-1.1153	0.032658999	0.000610353
BGI_novel_G000141	GP2	12.566	5.443	-1.11749	0.000000374	4.42E-10
ENSOCUG00000012833.4	SYT14_16	5.24	2.296	-1.12645	0.00000301	5.05E-09
ENSOCUG00000037634.1	SYN、PARP、DNAH、GNAO, G-ALPHA-O	3.363	1.406	-1.12707	0.004461015	0.0000361
ENSOCUG00000002973.4	PTCH1	18.79	7.736	-1.14217	0.001941239	0.0000115
ENSOCUG00000017153.4	KDM5, JARID1、CHFR、 CDHR5, MUCDHL	4.98	2.033	-1.14954	0.026784021	0.000442569
ENSOCUG00000007499.4	LMNB	19.603	7.696	-1.15462	0.026979528	0.000448461
ENSOCUG00000037255.1		6.336	2.433	-1.15945	0.045468713	0.001021356
ENSOCUG00000002814.4	TOP2	28.053	9.863	-1.17407	0.03600222	0.000708537
ENSOCUG00000005094.4	INHBB	37.58	15.273	-1.1912	0.0000395	9.35E-08
BGI_novel_G000128		4.726	1.843	-1.19872	0.003803529	0.0000283
ENSOCUG00000026133.3	BCAN	3.726	1.406	-1.2142	0.019302889	0.000260876
BGI_novel_G001667	GP2	1.743	0.656	-1.22312	0.016663958	0.000219362
ENSOCUG00000021324.3		8.13	2.473	-1.23195	0.011303324	0.0001267

ENSOCUG00000002818.3		4.07	1.07	-1.23662	0.022469452	0.00033278
ENSOCUG00000026281.3	PAK6	3.136	1.196	-1.24682	0.001770818	0.00000996
ENSOCUG00000009796.4	AQP9	2.66	1.02	-1.25881	0.000164865	0.000000545
ENSOCUG00000002719.3	EPN	1.503	0.506	-1.27802	0.049885168	0.001183524
ENSOCUG00000004877.4	ZMAT3、DHX58, LGP2	6.61	2.68	-1.28916	0.0000925	0.000000272
BGI_novel_G000587		6.666	2.213	-1.29025	0.044620725	0.000977193
ENSOCUG00000015611.4	FBLN1_2	10.04	3.46	-1.29251	0.016663958	0.000218445
ENSOCUG00000007663.4		11.643	3.91	-1.30155	0.046148437	0.001046537
ENSOCUG00000009507.4	ADAMTS16	0.596	0.196	-1.31461	0.044789739	0.00099192
ENSOCUG00000000370.4	ADAMTS8	3.346	1.1	-1.32231	0.03673801	0.000728594
ENSOCUG00000015436.4	ADAM22	13.486	4.733	-1.32539	0.004033688	0.0000308
ENSOCUG00000000187.3	CKAP4, CLIMP63	9.066	2.946	-1.32799	0.046535614	0.00106274
ENSOCUG00000013534.4	ENPP2	11.61	4.016	-1.32846	0.004033688	0.0000308
ENSOCUG00000017667.4	ATP1A	5.58	1.943	-1.33032	0.0000109	1.94E-08
ENSOCUG00000026214.3	RGS2	34.33	5.74	-1.33477	0.032215571	0.000592869
ENSOCUG00000000968.4	DES	65.063	24.283	-1.33504	4.85E-12	1.67E-15
ENSOCUG00000005167.4	ANGPT2、ANGPT1、ANGPT4、FCN	7.426	2.52	-1.33567	0.021545879	0.000304902
ENSOCUG00000035539.1	RP-S12e, RPS12	35.846	11.076	-1.35523	0.039967737	0.000835865
ENSOCUG00000008064.4	NEFH, NF-H、TCHH、 EPB41, 4.1R	1.193	0.45	-1.35616	0.041679659	0.000886061
ENSOCUG00000008628.4	PNPLA1	0.963	0.303	-1.35693	0.027539149	0.000459122
ENSOCUG00000009715.4	NEK2	6.52	1.966	-1.36375	0.047322293	0.001102156
BGI_novel_G000429		3.976	1.363	-1.36685	0.003129898	0.0000218
BGI_novel_G000261		40.62	14.13	-1.37676	0.0000259	5.23E-08
ENSOCUG00000032767.1	TAC2_3	20.423	7.076	-1.40104	0.000000565	6.97E-10
BGI_novel_G000650	GP2	53.19	17.023	-1.40312	0.005062422	0.0000444
BGI_novel_G000018	CSMD、TCHH、LPIN	1.803	0.596	-1.40954	0.00124721	0.00000634
ENSOCUG00000024805.3	COL5AS	1.016	0.3	-1.41786	0.030823325	0.000535844
ENSOCUG00000012176.3	UBE2C, UBC11	62.173	18.616	-1.42483	0.015660193	0.000193107
BGI_novel_G001507	GP2	1.073	0.333	-1.45928	0.002356196	0.0000153
ENSOCUG00000006396.4	CDC45	3.423	0.943	-1.47322	0.027562722	0.000460874
ENSOCUG00000030919.1	GP2	0.98	0.303	-1.47929	0.044789739	0.000987671
ENSOCUG00000005464.3	SLC3A1, RBAT	1.023	0.313	-1.54202	0.001283772	0.00000665
BGI_novel_G001732	KIF2_24, MCAK	15.903	4.12	-1.54511	0.0165515	0.000213078
BGI_novel_G001377	CENPT	1.416	0.356	-1.5545	0.047322293	0.001097997
BGI_novel_G002005		2.393	0.713	-1.5581	0.0000318	7.07E-08
BGI_novel_G001946		6.796	1.876	-1.57476	0.001954977	0.0000117
BGI_novel_G001107	ACIN1, ACINUS	1.033	0.216	-1.5986	0.044789739	0.000995896
BGI_novel_G002471	KRAB	1.493	0.4	-1.6002	0.002276297	0.0000145
BGI_novel_G000371		2.123	0.593	-1.63537	0.000066	0.000000176
BGI_novel_G000250	PNRC1、KRAB	126.25	37.236	-1.64176	2.29E-12	6.79E-16
BGI_novel_G000383	TCHH	1.286	0.223	-1.69988	0.03655558	0.000723034
BGI_novel_G000069		1.343	0.286	-1.70325	0.021575342	0.00030911
BGI_novel_G001421		1.846	0.34	-1.70922	0.031968085	0.000582648
BGI_novel_G002260		0.62	0.1	-1.7504	0.037067511	0.000740473
BGI_novel_G001420		1.166	0.24	-1.75293	0.008209306	0.0000846
BGI_novel_G001720	FOXP	7.353	1.496	-1.78372	0.005022481	0.0000431
ENSOCUG00000001046.4	IBSP	2.66	0.343	-1.78436	0.038414565	0.000774961
ENSOCUG00000009212.4	CD1	1.02	0.153	-1.78873	0.02656564	0.000429136
BGI_novel_G000586	GNAO, G-ALPHA-O、V2R、DNAH、CNGB3、SYN	11.053	1.936	-1.79252	0.021535869	0.000299552
ENSOCUG00000006728.4	TTN	0.89	0.196	-1.79655	0.001556482	0.00000829
ENSOCUG00000023032.3	NPC1L1	0.386	0.06	-1.80195	0.024341968	0.000386277
ENSOCUG00000021096.2	IGLL1, IGLL, CD179b、VPREB, CD179a	5.04	0.78	-1.8307	0.02656564	0.000430811
BGI_novel_G000372	E4.2.1.2B, fumC, FH、FBXL13、SIAH2、KRAB	15.506	1.863	-1.84882	0.030823325	0.000536679
ENSOCUG00000001190.4	GFRA1, GDNFRA	1.946	0.316	-1.85189	0.011556794	0.000131107
ENSOCUG00000031562.1	DHX15, PRP43	1.2	0.11	-1.88154	0.034163514	0.000653815
BGI_novel_G001722	COX2	8105.706	186.69	-1.89381	0.031020826	0.000547768
ENSOCUG00000002057.4	KNG, BK	1.7	0.276	-1.89522	0.007362204	0.0000737
ENSOCUG00000034802.1	SYN、DNAH、PARP、GNAO, G-ALPHA-O、CLCA3_4	0.36	0	-1.89936	0.030923891	0.000539955
ENSOCUG00000029605.2		14.413	0.843	-1.89947	0.031956806	0.000575366
ENSOCUG00000016287.4	ALB	1.193	0.123	-1.91115	0.024341968	0.000385142
BGI_novel_G000613		1.46	0.173	-1.94052	0.017521563	0.000234998
ENSOCUG00000031107.1	WIPF、CC2D2A、VPS16、SPTB	0.853	0	-1.9612	0.023515792	0.00036252
ENSOCUG00000035358.1	VPREB, CD179a	3.27	0.37	-1.99012	0.012776921	0.00014873
BGI_novel_G001756	COL7A	1.513	0.26	-2.04405	0.000216435	0.000000769
ENSOCUG00000035196.1	VPREB, CD179a	5.203	0.523	-2.12665	0.003343611	0.0000237
BGI_novel_G001736	CD2BP2, PPP1R59	2.656	0.356	-2.14888	0.001681298	0.00000929
ENSOCUG00000027394.3	GLL1, IGLL, CD179b、VPREB, CD179a、LPCAT4, AGPAT	10.063	0.916	-2.20869	0.003000842	0.0000206
BGI_novel_G001069		4.083	0.38	-2.42591	0.000216435	0.000000761
ENSOCUG00000039107.1	COX20	28.546	2.84	-2.50644	0.0000395	9.11E-08
ENSOCUG00000026795.1	MRPS33	8.933	0	-2.63377	0.000426838	0.00000174
ENSOCUG00000026406.2	BTN, CD277	2.566	0.156	-2.67552	0.0000708	0.000000192
ENSOCUG00000036212.1	CORO2	23.353	1.47	-2.70967	0.0000262	5.55E-08
ENSOCUG00000025405.2	GDI1_2	30.196	0.08	-3.19301	0.00000378	6.53E-09
ENSOCUG00000037309.1	WIPF、CC2D2A、VPS16、SPTB	3.566	0	-3.72363	2.53E-09	1.62E-12
BGI_novel_G000230	MTG1	1.13	0	-3.76951	7.76E-10	4.21E-13
BGI_novel_G002156	WNK, PRKWNK、 ELN	0.58	0	-3.7945	7.64E-10	3.77E-13
ENSOCUG00000003584.2	trxA	42.243	0	-5.1133	1.25E-22	1.85E-26
BGI_novel_G000231	MTG1	17.993	0	-7.28053	1.37E-62	6.75E-67
